# A Smartwatch or Just a Watch? A Validation Study of the Smartwatch KC08 for Measuring Blood Pressure

**DOI:** 10.3390/s25123793

**Published:** 2025-06-18

**Authors:** Susana López-Ortiz, Celia García-Chico, Lisa Musso-Daury, Sara González-Ustio, Saúl Peñín-Grandes, José Pinto-Fraga, Sergio Maroto-Izquierdo, Alejandro Santos-Lozano

**Affiliations:** 1 i+HeALTH Strategic Research Group, Department of Health Sciences, Miguel de Cervantes European University, 47012 Valladolid, Spain; 2 Physical Activity and Health Research Group (“PaHerg”), Research Institute of Hospital “12 de Octubre” (“Imas12”), 28041 Madrid, Spain

**Keywords:** wearable device, digital health, validation, photoplethysmography

## Abstract

(1) Background: The use of wearable cuffless devices has emerged as an out-of-office blood pressure (BP) monitor device to improve the management of hypertension. We aimed to validate a new, low-cost smartwatch for the measurement of BP and, secondarily, to assess its reliability for the measurement of blood oxygen saturation (SpO_2_) and heart rate. (2) Methods: We compared 1000 pairs of measurements (n = 100) acquired by a smartwatch (KC08) with those measured by a reference device. (3) Results: A total of 100 participants (41 ± 18 years) completed the measurements. The values for the systolic and diastolic BP, heart rate and SpO_2_ measured with the Smartwatch KC08 and the reference devices differed significantly (*p* < 0.05). In addition, the Smartwatch KC08 showed a low variability and poor reliability for all the assessed outcomes except for the heart rate. Moreover, no significant heteroscedasticity was observed for the heart rate measured by the Smartwatch KC08 and the reference sphygmomanometer. (4) Conclusions: The main findings of the present study suggest that the Smartwatch KC08 is not yet suitable for daily clinical practice, although it showed low intra-subject variability and reliability for the resting heart rate.

## 1. Introduction

The measurement of blood pressure (BP) is one of the most common clinical tools used to determine an individual’s cardiovascular status and predict future cardiovascular events [1]. High blood pressure, or hypertension, and pre-hypertension are important risk factors that contribute to the development of cardiovascular and cerebrovascular disease and are becoming the leading risk factors of more than 10 million deaths worldwide each year [2].

Adequate BP monitoring and maintenance of BP levels below target levels are critical for improving cardiovascular prognoses [3]. In recent years, ambulatory BP measurement has become an essential component of BP control, as evidenced by recent guidelines recommending its use to optimize the treatment and follow-up of people with hypertension [3,4]. Similarly, 24h ambulatory BP measurement and home BP measurement are acceptable methods for assessing hemodynamic stress in hypertensive patients outside the office [5]. The number of measurements that can be recorded with ambulatory and home monitoring is limited; clinically valid readings can only be obtained from blood pressure charts recorded by automated sphygmomanometers. This limitation emphasizes the need to develop wearable devices that allow for continuous measurement without a pressure cuff [5]. In this context, mobile health technologies (mHealth) have proven to be a promising tool for supporting blood pressure monitoring, improving adherence and increasing patient engagement through the integration of wearable devices with digital platforms and mobile applications [6,7]. The use of these new wearable technologies, such as smartwatches, facilitates monitoring during daily activities and improves adherence to therapy [8]. In addition, cuffless technology could solve the problems related to cuff intolerance and incorrect cuff application. However, there are currently still significant limitations to these devices, with the main ones being the measurement variability and the high economic cost of this technology [8]. In fact, cost remains a major barrier—high-end wearables are often prohibitively expensive for many users, highlighting the need for more affordable smartwatch alternatives [9]. Another concern is that once a new device enters the market people may use it for self-monitoring without proper validation or interpretation by clinicians [10].

Cuffless devices use new approaches, such as photoplethysmography (PPG), bioimpedance and mobile phone sensors to estimate the BP from the pulse transit time (PTT) or pulse wave velocity (PWV) [11,12]. The use of PPG signals has become a promising alternative to cuff-based BP devices. This technology uses infrared light to measure the volumetric changes in the vasculature [13].

In 2017, the American College of Cardiology (ACC), together with the American Heart Association (AHA), established guidelines recommending the use of validated wearable devices for BP assessment [4]. The KC08 Smartwatch represents a new, low-cost, non-validated and commercially available device that could be a cheaper alternative to validated devices, such as the Huawei Watch D (HUAWEI Technologies Co. Ltd., Shenzhen, China) and Omron HEM-6410T-ZL (Omron Healthcare, Kyoto, Japan). Accordingly, the main objective was to validate the low-cost KC08 Smartwatch for the measurement of BP. The secondary aims were to assess its reliability for the measurement of SpO_2_ and heart rate.

## 2. Materials and Methods

This cross-sectional study was conducted in accordance with the Declaration of Helsinki. The protocol was approved by the Ethics Committee for Research with Medicines of the Valladolid Health Areas (PI-24-444-C) on 10 July 2024. The protocol was prospectively registered with ClinicalTrials.gov (NCT06627920). All the participants provided written informed consent.

### 2.1. Eligibility Criteria

Adults with or without diseases were recruited from July 2024 to November 2024. Participants were included in the study if they were ≥18 years old and able to understand the informed consent form. Participants were excluded from the study if they had a difference of ≥20 mmHg in their systolic and/or diastolic BP between their two arms, following the criteria from a previous study [10].

Before starting with the measurements, the subjects were checked for compliance with the visit conditions according to the AHA recommendations [4]: (i) an empty bladder, (ii) no smoking (in the previous 30 min), (iii) no coffee or any kind of stimulant drink (in the previous 30 min) and (iv) no physical activity (in the previous 30 min).

### 2.2. Measurements

All the measurements were performed under standardized conditions in a quiet room under white light (18 W/230 V/50–60 Hz) and at a stable temperature (22–24 °C). The participants sat in a comfortable chair with lumbar support for at least five minutes before starting the measurements. In accordance with the AHA recommendations [4], the participants were asked to place both feet flat on the floor without crossing their legs and to place their arm with the cuff at chest level on a table with their palm facing upwards. The participants were asked not to speak during this procedure to ensure accurate and reliable BP readings. A measurement was taken of both arms to select the arm with the higher reading for the subsequent analysis. For each outcome, ten repeated measurements were taken with one-minute intervals between readings. Following the consensus of the International Organization for Standardization (ISO) [14], the sequential validation method was used (i.e., reference BP measurement followed by test device measurement) on the same arm.

#### 2.2.1. Primary Outcome: Blood Pressure Measurement

The reference method measurements were performed with a validated automatic cuff-based upper-arm monitor, an Omron M7 (Omron Healthcare Co., Ltd., Kyoto, Japan). A Smartwatch KC08 (Shenzhen Leyingchuang Trading Co., Ltd., Dongguan, China) was used for the cuffless BP measurement at the wrist of the same arm using its integrated optical sensor (Tianyihexin HRS3300, 525 nm green LED, Nanjing Tianyihexin Electronics Ltd., Nanjing, China) to perform a pulse wave analysis based on the PPG signals. The smartwatch was positioned 1 cm distal to the radial styloid process on the selected wrist, with the wristband (circumference from 14 to 19.6 cm) individually adjusted to each participant’s wrist size to ensure firm contact with the skin while avoiding blood flow restriction to minimize the displacement of the sensor during data collection. No calibration settings or measurement algorithms are specified in the manufacturer’s manual.

#### 2.2.2. Secondary Outcomes: Heart Rate and Blood Oxygen Saturation Measurement

The heart rate and blood oxygen saturation (SpO_2_) were assessed using an FDA-approved Pulse Oximeter Beurer PO30 (Beurer GmbH, Mittelstand, Ulm, Germany), placed on the index and middle fingers on the side selected for BP monitoring. The automatic sphygmomanometer Omron M7 was also used for assessing the heart rate. The Smartwatch KC08 was used to assess both outcomes using its integrated optical sensor (PPG signal).

### 2.3. Statistical Analyses

The statistical analyses were performed using IBM SPSS Statistics v.23.0 for Windows (SPSS Inc., Chicago, IL, USA) and GraphPad Prism v.8.0.1 for Windows (GraphPad Software Inc., Boston, MA, USA). The statistical significance was set at a *p*-value ≤ 0.05.

The descriptive statistics for the quantitative variables were calculated as the means and standard deviation (SD). Given the sample size, a Kolmogorov–Smirnov test was applied to determine whether the data followed a normal distribution.

Inferential statistical analyses were performed to compare the measurements of the Smartwatch KC08 and the corresponding reference devices. As the data were non-parametric, a Wilcoxon signed-rank test was conducted to compare the systolic and diastolic BP values obtained by the smartwatch against those recorded by the Omron M7 sphygmomanometer, as well as to compare the SpO_2_ measurements of the smartwatch and the Beurer PO30 pulse oximeter. To evaluate the differences in heart rate, a Friedman test was applied, with the device type as the independent factor.

The level of agreement between the Smartwatch KC08 and the reference devices was evaluated using the following statistical methods. The two-way random intraclass correlation coefficient (ICC) was calculated to assess the reliability of the measurements across the devices. ICC values below 0.5, between 0.5 and 0.75, between 0.75 and 0.9 and above 0.90 indicated poor, moderate, good and excellent reliability, respectively [15]. Moreover, the coefficient of variation (CV), defined as the ratio of the SD to the mean [CV = (SD/mean) × 100], was calculated for each participant and expressed as the general mean value for each device. The intra-subject variability was considered as follows: >10%, high; 5–10%, acceptable; and <5%, low [16].

A Bland–Altman analysis [17] was conducted to assess the agreement between the Smartwatch KC08 and each reference device (Omron M7 or Beurer PO30), calculating the BIAS (mean difference), SD of the BIAS and the 95% limits of agreement (LoA). Additionally, a linear regression analysis was performed to examine potential heteroscedasticity, assessing the association between the differences in the device measurements and the mean values of the measurements.

Pearson’s correlations were conducted to analyze the association between the Smartwatch KC08 and the reference device. The Pearson’s r values were interpreted as follows: ≤0.10, negligible correlation; 0.10–0.39, weak correlation; 0.40–0.69, moderate correlation; 0.70–0.89, strong correlation; and ≥0.9, very strong correlation [18].

## 3. Results

### 3.1. Participants

**Figure 1:**
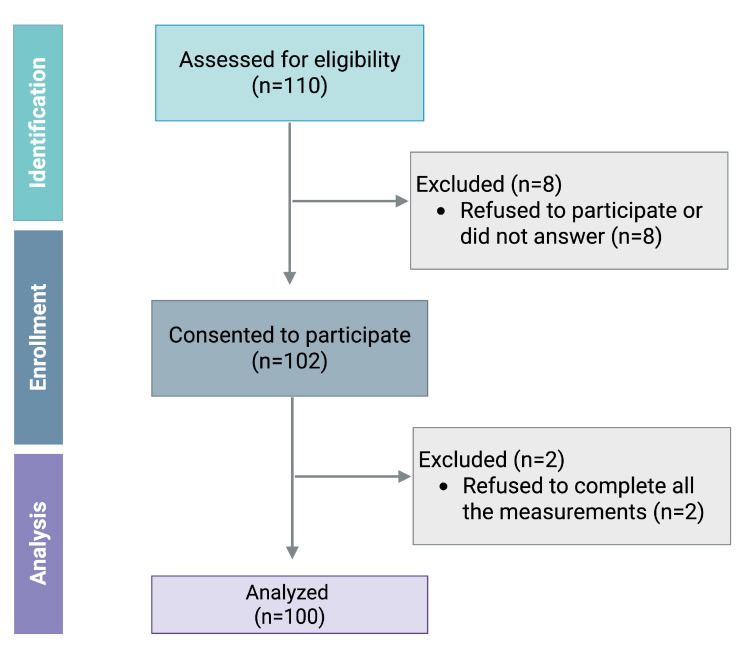
STROBE flow diagram for study participants.

One hundred and ten adults of Caucasian origin were assessed for eligibility in this study. Among them, 100 (41 ± 18 years) were included in the final analysis, excluding two who did not complete the measurements (see Figure 1). The characteristics and demographics of the participants are shown in Table 1.

### 3.2. Primary Outcome: Blood Pressure

The mean systolic BP measured with the smartwatch and the reference device differed significantly (112.33 ± 5.93 mmHg and 108.79 ± 15.17 mmHg, respectively). A similar difference was also found for the diastolic BP, with the smartwatch recording 72.45 ± 3.08 mmHg and the reference device 71.35 ± 9.70 mmHg (see Table 2).

The Smartwatch KC08 had a low intra-subject variability for both the systolic and diastolic BPs (CVs: 1.63% and 1.85%, respectively) (see Table 3). However, the ICC for both outcomes indicated poor reliability [ICC = 0.054 (95% CI: −0.071–0.164) for systolic BP and ICC = −0.027 (95% CI: −0.163–0.093) for diastolic BP].

**Figure 2:**
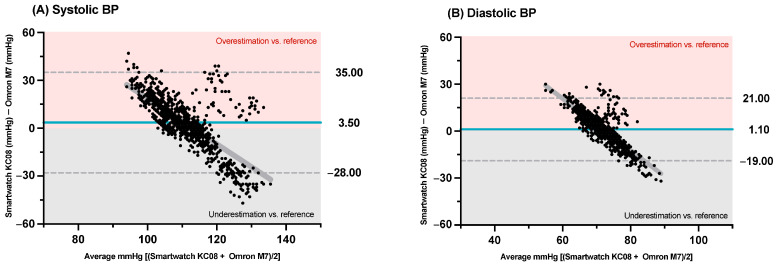
Bland–Altman plots of differences between (**A**) systolic blood pressure (BP) (mmHg) measured by Smartwatch KC08 and Omron M7 sphygmomanometer, and (**B**) diastolic BP (mmHg) measured by Smartwatch KC08 and Omron M7 sphygmomanometer reference device. Grey line represents heteroscedasticity trend.

In addition, the Bland–Altman analysis of the systolic BP (see Figure 2A) showed a BIAS between the difference in the BP from the Smartwatch KC08 and the reference device of 3.50 (95%LoA: −28 mmHg to 35 mmHg). For the diastolic BP (see Figure 2B), a BIAS between the difference in the BP predicted with the smartwatch and the reference device of 1.10 (95%LoA: −19 mmHg to 21 mmHg) was observed. There was significant heteroscedasticity in the results for both the systolic BP (r = 0.735; *p* < 0.001) and diastolic BP (r = 0. 815; *p* < 0.001). There was a non-significant correlation between the two devices for the measurement of the systolic BP (r = 0.041, *p* = 0.200) and diastolic BP (r = −0.023, *p* = 0.466).

### 3.3. Secondary Outcomes: Heart Rate and SpO_2_

The mean heart rate differed significantly between the three devices (see Table 2). However, no significant differences were observed (*p* = 0.123) between the smartwatch and the Omron M7 (69.89 ± 11.00 vs. 69.28 ± 11.07, respectively). In contrast, when comparing the smartwatch to the Beurer PO30 pulse oximeter, a significant difference was found (70.16 ± 11.46). Similarly, differences in the SpO_2_ measurements were noted between the smartwatch and the reference device (96.96 ± 0.84 vs. 96.60 ± 1.60, *p* < 0.001).

The Smartwatch KC08 had low and acceptable intra-subject variability for both the SpO_2_ and heart rate (CVs: 0.86 and 6.26, respectively) (see Table 3). The ICC indicated poor reliability for the SpO_2_ [ICC = 0.059 (95% CI: −0.060–0.166)] and excellent reliability for the heart rate [ICC = −0.947 (95% CI: 0.941–0.953)].

**Figure 3:**
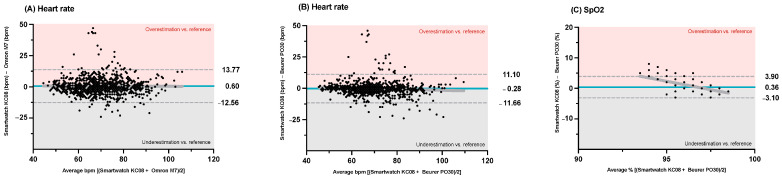
Bland–Altman plots of differences between (**A**) heart rate (beats per minute [bpm]) measured by Smartwatch KC08 and Omron M7 sphygmomanometer; (**B**) heart rate (bpm) measured by Smartwatch KC08 and Pulse Oximeter Beurer PO30; and (**C**) blood oxygen saturation (SpO_2_) measured by Smartwatch KC08 and Pulse Oximeter Beurer PO30. Grey line represents heteroscedasticity trend.

The Bland–Altman analysis of the heart rate (see Figure 3A,B) showed a BIAS of 0.60 (95%LoA: −12.56 bpm to 13.77 bpm) when comparing the smartwatch and the Omron M7 sphygmomanometer and a BIAS of −0.28 (95%LoA: −11.66 bpm to 11.10 bpm) when comparing the smartwatch and the Pulse Oximeter Beurer PO30. The Bland–Altman plots for the SpO_2_ (see Figure 3C) showed a BIAS of 0.36 (95%LoA: −3.10% to 3.90%). The heteroscedasticity was significant in the results for the heart rate measured by the Smartwatch KC08 and Pulse Oximeter Beurer PO30 (r = 0.01; *p* = 0.007) and for the SpO_2_ measured by the Smartwatch KC08 and Pulse Oximeter Beurer PO30 (r = 0.569; *p* < 0.001). No significant heteroscedasticity was observed for the heart rate measured by the Smartwatch KC08 and Omron M7 sphygmomanometer (r < 0.001; *p* = 0.759).

A strong and significant correlation was observed for the heart rate measurements between the Smartwatch KC08 and the Omron M7 sphygmomanometer (r = 0.815, *p* < 0.001) and between the Smartwatch KC08 and the Pulse Oximeter Beurer PO30 (r = 0.867, *p* < 0.001). The heart rate assessment correlation between the Omron M7 sphygmomanometer and the Pulse Oximeter Beurer PO30 was also strong and significant (r = 0.890; *p* < 0.001). For the SpO_2_, there was a negligible non-significant correlation between the two devices (r = 0.039; *p* = 0.224).

## 4. Discussion

The aim of this cross-sectional study was to validate a new, low-cost smartwatch for the measurement of BP and, secondarily, for the measurement of SpO_2_ and heart rate. The main findings of our study are that the Smartwatch KC08 is not a reliable device for the monitoring of either BP or SpO_2_, but has good reliability for assessing heart rate.

The 2024 European Society of Cardiology (ESC) Guidelines [19], along with the 2017 ACC/AHA Guidelines [4], recommend out-of-office BP measurements as they provide better prognostic information than measuring BP in a clinic alone. An emerging technology that relies on optical sensors to monitor BP throughout the day is wearable, cuffless devices, such as smartwatches [11,20]. This technology may improve the awareness and adherence of patients with hypertension, but some potential limitations should be considered [20]. Several devices currently on the market have not been validated or their accuracy has not been adequately assessed [11]. These include the Smartwatch KC08, which has shown a low correlation with the reference device for both systolic and diastolic BP measurements and a low CV, which in this case might suggest that it lacks sufficient sensitivity to detect small fluctuations. In comparison, the validated Omron M7 automated device also has a low CV, but it is still higher than that of the Smartwatch KC08. Notably, the algorithms used by the Smartwatch KC08 to measure the outcomes assessed have not been openly disclosed and a single account was used for all the measurements, which could limit the results if the algorithm uses an adjustment for an individual’s baseline data and demographics. These results suggest that smartwatch-based BP measurement is not ready for clinical use, as previously shown for other wrist-worn devices, such as the Samsung Galaxy Watch Active 2 (Samsung Electronics Co., Ltd., Suwon-si, Republic of Korea) [10] and Everlast TR10 Smartwatch (Everlast, Hong Kong, China) [21]. However, these results differ from those of other smartwatches, such as the Huawei Watch D [22,23], the Omron HEM-6410T-ZM [24] and the Omron HEM-6410T-ZL [24], which integrate oscillometric technology for blood pressure measurement. These devices are equipped with a small inflatable cuff on the wristband that inflates for a more accurate measurement, similar to that of a traditional blood pressure monitor. This approach is more reliable than measurements with optical sensors, although it is still less practical due to its size and the need for regular calibration [24].

The resting heart rate measurements with the Smartwatch KC08 proved to be reliable (ICC= 0.898; 95% CI: 0.884–0.910, *p* < 0.001) and accurate when compared to the automatic blood pressure monitor. These findings are consistent with previous studies that have evaluated other smartwatches, which have shown heart rate measurements with this type of device to have the highest correlation with validated medical equipment [25,26].

The results for the SpO_2_ are similar to those for the blood pressure, where the device did not show sufficient consistency in measurement. Although the pulse oximeters used in clinical settings are also based on optical technology, smartwatch sensors lack the precision required for reliable readings. The tests performed showed that the SpO_2_ values determined by the smartwatch tended to vary widely and showed significant differences compared to the pulse oximeters, which is consistent with the results of a study using the Everlast TR10 smartwatch [21]. Our results appear to differ from those obtained with other smartwatches [27,28,29], which appear to have high reliability and accuracy for measuring SpO_2_. These differences between the devices can be attributed to several factors, such as the placement of the device on the wrist, as the variability in blood flow is higher there than in the distal phalanx. Body temperature may also influence the measurements of both SpO_2_ and BP. On the other hand, external interferences, such as ambient light, skin pigmentation and user movement during the measurement, can affect the accuracy of watch sensors [27]. While smartphone and wrist-worn devices may offer a convenient solution for continuous monitoring at home, their limitations must be carefully considered for clinical practice, particularly when measurements are taken outside of controlled environments.

However, the current study is not without limitations. First, the study was conducted in a general Caucasian adult population, which may limit the applicability of the results to other populations (e.g., children, pregnancy, atrial fibrillation), including patients with cardiovascular disease. Notably, only five and six participants had a mean systolic BP value ≥ 140 mmHg and mean diastolic BP ≥ 85 mmHg, respectively, which is below the current ISO distribution recommendations for BP, which state that at least 20% of a sample should be above these values [14]. Considering the results obtained, future studies should investigate the reliability of and intra-subject variability in the Smartwatch KC08 in hypertensive individuals to ensure its applicability in clinical practice, since the algorithms used by the smartwatch to generate data are unknown. Moreover, all the outcomes were measured in an indoor and controlled environment in a seated position, so the reliability of the device during physical activities, such as walking and running, remains unknown and further research is needed to confirm its use. Although the Smartwatch KC08 appears to be a valid device for measuring and monitoring heart rate, its variability may still be influenced by exercise intensity and other potential cofounding factors.

## 5. Conclusions

The main findings of the present study suggest that the Smartwatch KC08 (a low-cost smartwatch) is reliable for measuring the resting heart rate but not for blood pressure and SpO_2_. Therefore, it may not provide clinically meaningful data and is not recommended for clinical use. Further research is needed to confirm the results in a more heterogeneous sample, including a sub-analysis of different special populations.

## Figures and Tables

**Table 1:** Descriptives characteristics of participants.

	Participants (n = 100)
Gender—n (% women)	62 (62%)
Caucasian—n (%)	100 (100%)
Age—years	40.94 ± 17.54
Weight—kg	69.46 ± 12.42
Height—m	1.68 ± 0.09
BMI—kg/m^2^	24.12 ± 4.37
Current smoking—n (%)	17 (17%)
Current pharmacological treatment—n (%)	37 (37%)

**Table 2:** Comparison of measurements between devices. Bpm, beats per minute; mmHg, millimeters of mercury. Data are presented as mean ± standard deviation (minimum–maximum range) and median [first–third quartile].

Outcome	Smartwatch KC08	Omron M7	Beurer PO30	*p*-Value
Systolic blood pressure (mmHg)	112.33 ± 5.93 (103–140)	108.79 ± 15.17 (71–153)	-	<0.001
	111.00 [109.00–114.00]	107.00 [98.25–116.00]	-	
Diastolic blood pressure (mmHg)	72.45 ± 3.08 (66–87)	71.35 ± 9.70 (40–105)	-	0.032
	72.00 [71.00–73.00]	71.00 [65.00–78.00]	-	
Heart rate (bpm)	69.89 ± 11.00 (46–112)	69.28 ± 11.07 (43–103)	70.16 ± 11.46 (43–112)	<0.001
	69.00 [62.00–77.00]	69.00 [62.00–76.00]	69.00 [63.00–77.00]	
Blood oxygen saturation (%)	96.96 ± 0.84 (94–99)	-	96.60 ± 1.60 (90–99)	<0.001
	97.00 [96.00–98.00]	-	97.00 [96.00–98.00]	

**Table 3:** Analysis of intra-subject variability and inter-device reliability of blood pressure, heart rate and blood oxygen saturation within and between devices. Bpm, beats per minute; CV, coefficient of variation; ICC, intraclass correlation coefficient; mmHg, millimeters of mercury.

Outcome	Smartwatch KC08 CV (%)	Omron M7 CV (%)	Beurer PO30 CV (%)	ICC (95% CI)	*p*-Value
Systolic blood pressure (mmHg)	1.63	4.07	-	0.054 (−0.071–0.164)	0.192
Diastolic blood pressure (mmHg)	1.85	4.87	-	−0.027 (−0.163–0.093)	0.663
Heart rate (bpm)	6.26	4.65	4.95	0.947 (0.941–0.953)	<0.001
Blood oxygen saturation (%)	0.86	-	0.70	0.059 (−0.060–0.166)	0.158

## Data Availability

Data will be made available upon reasonable request.

## Abbreviations

The following abbreviations are used in this manuscript:

ACC	American College of Cardiology
AHA	American Heart Association
BP	Blood pressure
bpm	Beats per minute
CI	Confidence interval
CV	Coefficient of variation
ESC	European Society of Cardiology
ISO	International Organization for Standardization
LoA	Limits of agreement
mmHg	Millimeters of mercury
SD	Standard deviation
SpO_2_	Blood oxygen saturation
PPG	Photoplethysmography
PPT	Pulse transit time
PWV	Pulse wave velocity

## References

[1] Jose A.P., Awasthi A., Kondal D., Kapoor M., Roy A., Prabhakaran D. (2019). Impact of repeated blood pressure measurement on blood pressure categorization in a population-based study from India. J. Hum. Hypertens..

[2] Boateng E.B., Ampofo A.G. (2023). A glimpse into the future: Modelling global prevalence of hypertension. BMC Public Health.

[3] Williams B., Mancia G., Spiering W., Agabiti Rosei E., Azizi M., Burnier M., Clement D.L., Coca A., de Simone G., Dominiczak A. (2018). 2018 ESC/ESH Guidelines for the management of arterial hypertension. Eur. Heart J..

[4] Whelton P.K., Carey R.M., Aronow W.S., Casey D.E., Collins K.J., Dennison Himmelfarb C., DePalma S.M., Gidding S., Jamerson K.A., Jones D.W. (2018). 2017 ACC/AHA/AAPA/ABC/ACPM/AGS/APhA/ASH/ASPC/NMA/PCNA Guideline for the Prevention, Detection, Evaluation, and Management of High Blood Pressure in Adults: A Report of the American College of Cardiology/American Heart Association Task Force on Clinical Practice Guidelines. Hypertension.

[5] Konstantinidis D., Iliakis P., Tatakis F., Thomopoulos K., Dimitriadis K., Tousoulis D., Tsioufis K. (2022). Wearable blood pressure measurement devices and new approaches in hypertension management: The digital era. J. Hum. Hypertens..

[6] Margolis K.L., Asche S.E., Bergdall A.R., Dehmer S.P., Groen S.E., Kadrmas H.M., Kerby T.J., Klotzle K.J., Maciosek M.V., Michels R.D. (2013). Effect of home blood pressure telemonitoring and pharmacist management on blood pressure control: A cluster randomized clinical trial. JAMA.

[7] McManus R.J., Little P., Stuart B., Morton K., Raftery J., Kelly J., Bradbury K., Zhang J., Zhu S., Murray E. (2021). Home and Online Management and Evaluation of Blood Pressure (HOME BP) using a digital intervention in poorly controlled hypertension: Randomised controlled trial. BMJ.

[8] Bradley C.K., Shimbo D., Colburn D.A., Pugliese D.N., Padwal R., Sia S.K., Anstey D.E. (2022). Cuffless Blood Pressure Devices. Am. J. Hypertens..

[9] Piwek L., Ellis D.A., Andrews S., Joinson A. (2016). The Rise of Consumer Health Wearables: Promises and Barriers. PLoS Med..

[10] Falter M., Scherrenberg M., Driesen K., Pieters Z., Kaihara T., Xu L., Caiani E.G., Castiglioni P., Faini A., Parati G. (2022). Smartwatch-Based Blood Pressure Measurement Demonstrates Insufficient Accuracy. Front. Cardiovasc. Med..

[11] Islam S.M.S., Chow C.K., Daryabeygikhotbehsara R., Subedi N., Rawstorn J., Tegegne T., Karmakar C., Siddiqui M.U., Lambert G., Maddison R. (2022). Wearable cuffless blood pressure monitoring devices: A systematic review and meta-analysis. Eur. Heart J. Digit. Health.

[12] Moon J.H., Kang M.K., Choi C.E., Min J., Lee H.Y., Lim S. (2020). Validation of a wearable cuff-less wristwatch-type blood pressure monitoring device. Sci. Rep..

[13] Man P.K., Cheung K.L., Sangsiri N., Shek W.J., Wong K.L., Chin J.W., Chan T.T., So R.H. (2022). Blood Pressure Measurement: From Cuff-Based to Contactless Monitoring. Healthcare.

[14] Stergiou G.S., Alpert B., Mieke S., Asmar R., Atkins N., Eckert S., Frick G., Friedman B., Grassl T., Ichikawa T. (2018). A universal standard for the validation of blood pressure measuring devices: Association for the Advancement of Medical Instrumentation/European Society of Hypertension/International Organization for Standardization (AAMI/ESH/ISO) Collaboration Statement. J. Hypertens..

[15] Koo T.K., Li M.Y. (2016). A Guideline of Selecting and Reporting Intraclass Correlation Coefficients for Reliability Research. J. Chiropr. Med..

[16] Hajj-Boutros G., Landry-Duval M.A., Comtois A.S., Gouspillou G., Karelis A.D. (2023). Wrist-worn devices for the measurement of heart rate and energy expenditure: A validation study for the Apple Watch 6, Polar Vantage V and Fitbit Sense. Eur. J. Sport Sci..

[17] Bland J.M., Altman D.G. (1986). Statistical methods for assessing agreement between two methods of clinical measurement. Lancet.

[18] Schober P., Boer C., Schwarte L.A. (2018). Correlation Coefficients: Appropriate Use and Interpretation. Anesth. Analg..

[19] McEvoy J.W., McCarthy C.P., Bruno R.M., Brouwers S., Canavan M.D., Ceconi C., Christodorescu R.M., Daskalopoulou S.S., Ferro C.J., Gerdts E. (2024). 2024 ESC Guidelines for the management of elevated blood pressure and hypertension. Eur. Heart J..

[20] Stergiou G.S., Mukkamala R., Avolio A., Kyriakoulis K.G., Mieke S., Murray A., Parati G., Schutte A.E., Sharman J.E., Asmar R. (2022). Cuffless blood pressure measuring devices: Review and statement by the European Society of Hypertension Working Group on Blood Pressure Monitoring and Cardiovascular Variability. J. Hypertens..

[21] Hahnen C., Freeman C.G., Haldar N., Hamati J.N., Bard D.M., Murali V., Merli G.J., Joseph J.I., van Helmond N. (2020). Accuracy of Vital Signs Measurements by a Smartwatch and a Portable Health Device: Validation Study. JMIR Mhealth Uhealth.

[22] Zhang W., Zhou Y.N., Zhou Y., Wang J.G. (2022). Validation of the watch-type HUAWEI WATCH D oscillometric wrist blood pressure monitor in adult Chinese. Blood Press. Monit..

[23] Yi L., Lv Z.H., Hu S.Y., Liu Y.Q., Yan J.B., Zhang H., Li H.B., Chen Q., Li Y.Y., Jiang Y.F. (2022). Validating the accuracy of a multifunctional smartwatch sphygmomanometer to monitor blood pressure. J. Geriatr. Cardiol..

[24] Kuwabara M., Harada K., Hishiki Y., Kario K. (2019). Validation of two watch-type wearable blood pressure monitors according to the ANSI/AAMI/ISO81060-2:2013 guidelines: Omron HEM-6410T-ZM and HEM-6410T-ZL. J. Clin. Hypertens..

[25] Montalvo S., Martinez A., Arias S., Lozano A., Gonzalez M.P., Dietze-Hermosa M.S., Boyea B.L., Dorgo S. (2023). Commercial Smart Watches and Heart Rate Monitors: A Concurrent Validity Analysis. J. Strength Cond. Res..

[26] Duking P., Giessing L., Frenkel M.O., Koehler K., Holmberg H.C., Sperlich B. (2020). Wrist-Worn Wearables for Monitoring Heart Rate and Energy Expenditure While Sitting or Performing Light-to-Vigorous Physical Activity: Validation Study. JMIR Mhealth Uhealth.

[27] Zeng Z., Li L., Hu L., Wang K., Li L. (2024). Smartwatch measurement of blood oxygen saturation for predicting acute mountain sickness: Diagnostic accuracy and reliability. Digit. Health.

[28] Lauterbach C.J., Romano P.A., Greisler L.A., Brindle R.A., Ford K.R., Kuennen M.R. (2021). Accuracy and Reliability of Commercial Wrist-Worn Pulse Oximeter During Normobaric Hypoxia Exposure Under Resting Conditions. Res. Q. Exerc. Sport.

[29] Pipek L.Z., Nascimento R.F.V., Acencio M.M.P., Teixeira L.R. (2021). Comparison of SpO(2) and heart rate values on Apple Watch and conventional commercial oximeters devices in patients with lung disease. Sci. Rep..

